# The Electronic and Optical Properties of InSe-GeTe Heterobilayer via Applying Biaxial Strain

**DOI:** 10.3390/nano9121705

**Published:** 2019-11-28

**Authors:** Guofeng Yang, Rui Sun, Yan Gu, Feng Xie, Yu Ding, Xiumei Zhang, Yueke Wang, Bin Hua, Xianfeng Ni, Qian Fan, Xing Gu

**Affiliations:** 1 School of Science, Jiangnan University, Wuxi 214122, China; 6171201003@stu.jiangnan.edu.ch (R.S.); 6171203001@stu.jiangnan.edu.cn (Y.G.); 6181203010@stu.jiangnan.edu.cn (Y.D.); xiumeizhang@jiangnan.edu.cn (X.Z.); ykwang@jiangnan.edu.cn (Y.W.); 2 Nanjing Zike Optoelectronic Co., Ltd, Nanjing 211112, China; fxie@foxmail.com; 3 Institute of Next Generation Semiconductor Materials, Southeast University, Suzhou 215123, China; 103200046@seu.edu.cn (B.H.); 103200036@seu.edu.cn (X.N.); 103200035@seu.edu.cn (Q.F.); 103200014@seu.edu.cn (X.G.)

**Keywords:** InSe-GeTe heterobilayer, electronic structure, biaxial strain, absorption

## Abstract

A comprehensive insight into the electronic and optical properties of small-lattice-mismatched InSe-GeTe heterobilayer (HBL) is performed based on the density functional theory (DFT) with van der Waals corrections from first-principles perspective. The optimization of most stable geometric stacking mode for the InSe-GeTe HBL is demonstrated. In addition, it is found that the InSe-GeTe HBL forms a type-II heterostructure of staggered-gap band alignment, resulting in an indirect band gap of 0.78 eV, which could be employed as a separator for electron-hole pairs. Moreover, the influence of biaxial strain on the electronic and optical properties of the InSe-GeTe HBL are systematically explored by calculating the band structures, density of states (PDOS), electron density differences, and optical absorption spectra of InSe-GeTe HBL under compressive and tensile biaxial strains. The results indicate that the electronic structures and optical performance of InSe-GeTe HBL could be modulated by changing the biaxial strain conveniently. Our findings provide new opportunities for the novel InSe-GeTe HBL to be applied in the electronic and optoelectronic fields.

## 1. Introduction

**Figure 1:**
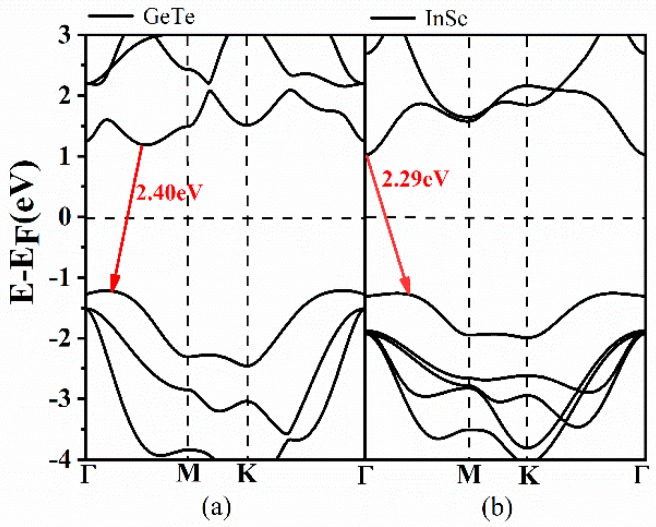
Calculated band structures of monolayer (ML) GeTe and InSe along symmetry directions of the Brillouin zone (Γ-M-K-Γ). The Fermi energy (E_F_) is set as zero, in order to facilitate the negative meaning of valence band and positive meaning of conduction band.

The promising and sustainable strategies of how to improve the utilization of solar energy has become a hot research topic, including solar cells, photo-degradation of contaminants, and so on [1]. Among them, appropriate band structure and advantageous charge separation are extremely important for efficient use of solar energy. Unfortunately, the vast majority of electron-hole pairs generated by photo excitation usually readily recombine in single semiconductor materials, owing to the occupation of the same area in space. In addition, a host of semiconductor materials only absorb a small proportion solar spectrum ranging in the visible light or ultraviolet wavelength attributed to their specific band gaps [2]. In the past years, there was still no effective strategy to work out an appropriate single material system to satisfy these requirements. Until 2013, the advent of two-dimensional (2D) Van der Waals (vdW) heterostructures, formed by stacking different 2D atomic semiconductor materials on top of each other, opened up new strategies for researchers to modulate 2D material properties to achieve superior optoelectronic device performance [3]. Moreover, based on previous reports, type-II staggered-gap structure could achieve effective spatial separation of photo-generated hole-electron pairs, breaking the limitations of an individual material. Moreover, the band structure as well as optical performance can also be controlled by applying vertical or in-plane stress even an external electric field [4]. For example, a series of theoretical studies [5,6,7] reported that graphene/silicone heterobilayer (HBL) exhibited stronger optical absorption than silicene and graphene monolayers. More recently, germanium monochalcogenides (GeX, X = S, Se, Te) arouse widespread attention in scientific community, because of their excellent mechanical flexibility, high carrier mobility, suitable band structures as well as inherent dipoles with large difference in electronegativity [8,9,10]. Particularly, layered Germanium telluride (GeTe), has been widely investigated in various fields due to its predominate semiconducting and ferroelectric nature, such as Li-ion batteries [11] and photocatalytic water splitting [12]. Qiao et al. predicted that the 2D monolayer (ML) GeTe could be facilely exfoliated from its bulk phase and exhibited excellent optical properties in the visible light wavelength [12]. A band gap of 2.40 eV for ML GeTe was also obtained theoretically by our first-principles calculations in this work, (shown in Figure 1), which coincides well with the previous results. Zhang et al. also indicated ML GeTe would be promising materials for applications in optoelectronics by a combination of experiment and theory, especially in the ultraviolet region [13]. On the other hand, 2D indium selenide (InSe) with high environmental stability and high carrier mobility (~10^3^ cm^2^·V^−1^·s^−1^), was successfully produced experimentally. Particularly, it is noted that the lattice mismatch between the ML GeTe and InSe is quite small, less than 1%, which could facilitate the formation of InSe-GeTe HBL by chemical or physical synthesis and growth. More importantly, it was easy to tune the electronic structures by exerting external electric field or strain upon their separate individuals [14]. Therefore, it is highly ideal and desirable to design proper InSe-GeTe heterostructure for potential applications.

In this work, in order to excavate the potential of 2D InSe-GeTe HBL. We first built six different stacking models of InSe-GeTe heterostructure by horizontal sliding or vertical turning, and confirm the most stable InSe-GeTe heterostructure by first-principles calculations. The InSe-GeTe HBL demonstrated a type-II band alignment with electrons and holes localized in the ML InSe and GeTe, respectively, which promoted efficient separation of electron-hole pairs. Then, the modulation of band structures and optical characteristics of the InSe-GeTe HBL were systematically investigated by applying biaxial strain. The present work would provide great opportunities for the potential applications of InSe-GeTe heterostructure in nano-electronic devices.

## 2. Calculation Methods 

The geometric structure optimizations and calculations were carried out by first-principles based on DFT with the Linear combination of atomic orbitals (LCAO) model in QuantumATK O-2018.06 code [15,16,17]. Perdew-Burke-Ernzerhof (PBE), which is important in the generalized gradient approximation (GGA) [18], is utilized to represent exchange-related potentials. Grimme D3 was adopted to account properly for van der Waals interactions between InSe-GeTe HBL. These corrections attempted to describe effect on the DFT mean-field effective potential [19]. An 8 × 8 × 1 Monkhorst-Pack k point mesh was sampled in the Brillouin zone, with the mesh cutoff density set as 100 Hartree, which can obtain great convergence results. All of the atomic positions were and optimized and relaxed until the forces tolerance per atom were smaller than 0.01, while the stress error tolerance was less than 10^−5^ eV. The length of vacuum zone was set to no less than 20 Å along the c-direction, for the purpose of avoiding spurious interactions between the adjacent slabs. Table 1 summaries the first-principles calculation parameters of InSe-GeTe HBL.

In order to figure out the optical characteristics including dielectric function and absorption, dipole approximation with Fermi golden rule is used to determine the dielectric function dependent to frequency. The dielectric function could be expressed by $\varepsilon \left(\omega \right)=\varepsilon_{1}\left(\omega \right)+i\varepsilon_{2}\left(\omega \right)$ [20], where $\varepsilon_{1}\left(\omega \right)$ is the real part and $\varepsilon_{2}\left(\omega \right)$ is the imaginary part. Here, the imaginary part is expressed as following [21]:

(1) $$\varepsilon_{2}\left(\hbar \omega \right)=\frac{4\pi^{2}e^{2}}{{\varphi \varepsilon }_{0}}\underset{c,v,k}{\sum }{\left|\psi_{k}^{c}\left|u\cdot r\right|\psi_{k}^{v}\right|}^{2}\delta \left(E_{k}^{c}-E_{k}^{v}-E\right)$$

where ω, $\varepsilon_{0},\mathrm{and}$ φ,  represent the photon frequency, vacuum dielectric constant, and crystal volume, respectively. The subscripts of c and v are the conduction and valence states associated with the energies $E_{k}^{c}$ and $E_{k}^{v}$, respectively. u defines the vector of polarization for the incident electric field. The momentum operator is described by u×r. The real part of $\varepsilon_{1}\left(\omega \right)$ could be obtained from $\varepsilon_{2}\left(\omega \right)$ by the well-known Kramers-Kronig relation:

(2) $$\varepsilon_{1}\left(\omega \right)=1+\frac{2}{\pi }Ρ\int_{0}^{\infty }\frac{\varepsilon_{2}\left(\omega^{\prime }\right)\omega^{\prime }}{\omega^{\prime 2}-\omega^{2}+i\eta }d\omega^{\prime }$$

where P represents the principle value of integrals, and η is the refractive index. Accordingly, the absorbance (α) would be calculated by the expression as following:

(3) $$\alpha \left(\omega \right)=\sqrt{2}\omega {\left[\sqrt{\varepsilon_{1}{}^{2}\left(\omega \right)+\varepsilon_{2}{}^{2}\left(\omega \right)}-\varepsilon_{1}\left(\omega \right)\right]}^{1/2}$$

## 3. Results and Discussion

### 3.1. Geometric Structures of InSe-GeTe HBL

**Figure 2:**
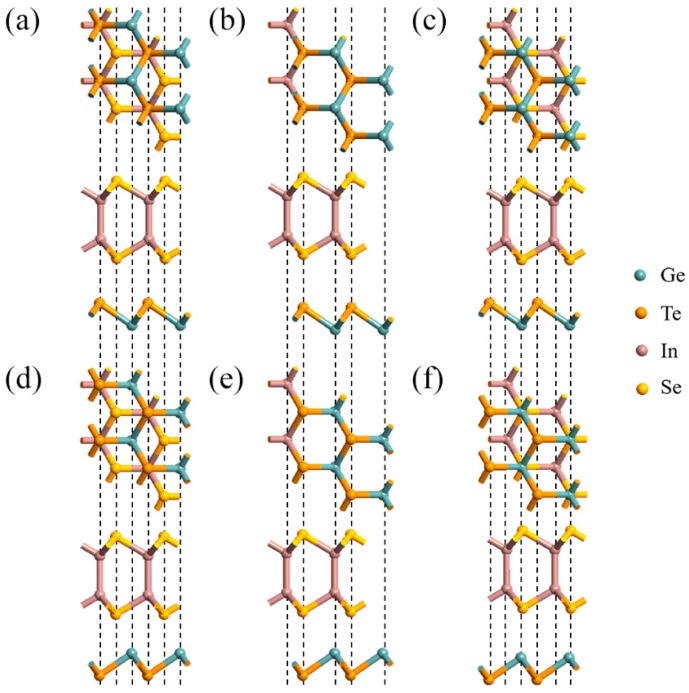
(**a**–**f**) Top (upper) and side (lower) views of the six different stacking types for Indium selenide-Germanium telluride (InSe-GeTe) heterobilayer (HBL).

We firstly explore the atomic geometries of the separate InSe and GeTe monolayers. The optimized lattice constants of the free-standing ML InSe and GeTe are 4.00 and 3.96 Å, respectively. While the bond lengths of In-Se, In-In, and Ge-Te are 2.67 Å, 2.81 Å, and 2.77 Å, respectively, which agrees well with the previous reported results [22,23,24], verifying the reliability of the optimized structures for both two pristine monolayers. When constructing InSe-GeTe HBL, a minimal 1 × 1 supercell is adopted, because the lattice mismatch between InSe and GeTe monolayers is quite small (~0.67%), and allows for constructing the stable stacking pattern of InSe/GeTe HBL. In this work, six different types of geometric stacking named I~VI are considered, which can be converted by horizontal sliding or vertical turning, as shown in Figure 2. As for I/IV-stacking shown in Figure 2a,d, it is observed that one Ge atom in the supercell is designed to locate on the hollow site above or below a hexagonal InSe center. For II/V-stacking (see Figure 2b,e), InSe and GeTe monolayers are perfectly constructed without any mismatch in the x-y plane. Whereas for III/VI-stacking shown in Figure 2c,f, Ge atoms are set to sit on the below or top of the Se atoms. To confirm the stability of the stacking systems, the binding energies, interlayer spacings, and band gaps of the six different InSe-GeTe stacking structures are calculated and listed in Table 2. The binding energy of E_b_ is defined as a criteria for the strength of interaction between HBL, and can be expressed as [25,26]:

E_b_ = [E_InSe-GeTe_ − (E_InSe_ + E_GeTe_)]/A (4)

where E_InSe-GeTe_ is the total energy of optimized InSe-GeTe HBL. E_InSe_ and E_GeTe_ denote the total energies of separate ML InSe and GeTe, respectively. A represents the interfacial area. It can be concluded from Table 1 that the type-IV HBL exhibits the best stability among the six stacking structures, which exhibits the minimum negative E_b_ value and interfacial spacing. Therefore, it is convincingly to choose the type-IV HBL as an atomic construction to explore its electronic and optical properties.

### 3.2. Electronic and Optical Properties of InSe-GeTe HBL

**Figure 3:**
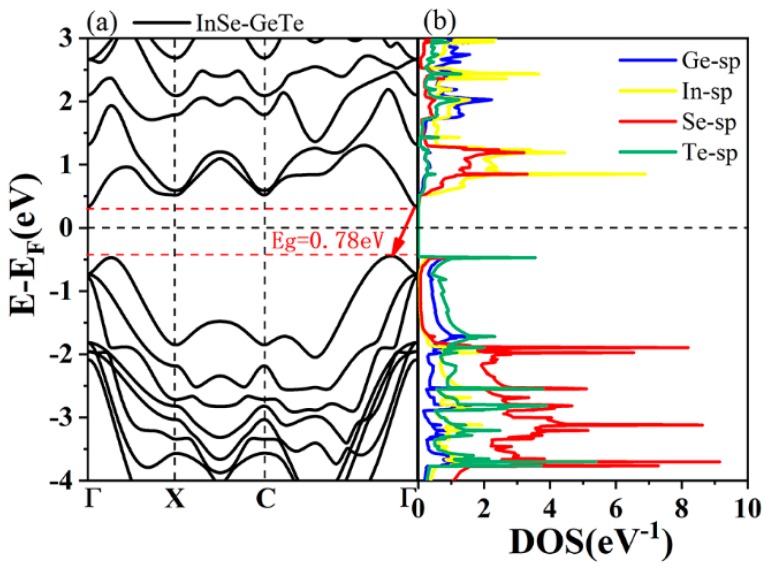
(**a**) Band structure of the InSe-GeTe HBL. (**b**) The density of states for the InSe-GeTe HBL (Fermi level is indicated by black dashed line).

Before investigating the energy band structure of the type-IV InSe-GeTe HBL, it is reasonable to firstly confirm the corresponding structures of both separate of InSe and GeTe monolayers. As plotted in Figure 1, the calculated band gap values of ML GeTe and InSe are 2.40 eV and 2.29 eV, respectively. After constructing the HBL of ML InSe and GeTe, the simulated band structures of the HBL are obtained in Figure 3a. It is observed that the InSe-GeTe HBL reveals an indirect band gap of 0.78 eV with its conductive band minimum (CBM) located around the Γ point, and valence band maximum (VBM) situated between the C and Γ points. Additionally, the density of states (obtained by tetrahedron integration) for InSe-GeTe HBL are plotted in Figure 3b, from which we can conclude that the individual GeTe and InSe primarily contribute to the VBM and CBM of InSe-GeTe HBL, respectively.

**Figure 4:**
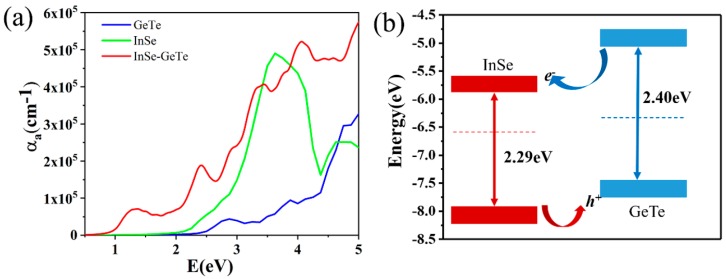
(**a**) Optical absorption coefficients of separate ML InSe (green line), GeTe (blue line), and InSe-GeTe HBL (red line), respectively. (**b**) A schematic diagram with band alignment for InSe-GeTe HBL.

To further explore the optical performance of the InSe-GeTe HBL, we calculate the absorption spectra of the separate ML InSe, ML GeTe, and InSe-GeTe HBL, as depicted in Figure 4a. It is noted that ML GeTe exhibits only a small absorption peak located in the visible light wavelength, while a narrower absorption peak located around 3.5 eV for ML InSe is observed. However, when the InSe-GeTe HBL structure forms, the absorption spectra of the HBL indicates a wider absorption edge extending from visible to infrared light region compared to that of the separate individual monolayers, which would be attributed to the hybridization of electronic states between the two monolayers. Moreover, a significant enhancement of the absorption intensity for InSe-GeTe HBL compared to that of the separate monolayers is probably caused by the interlayer coupling between InSe and GeTe monolayers. Figure 4b illustrates the band alignment of the InSe-GeTe HBL. The CB and VB energy levels of ML GeTe are higher than the corresponding energy bands in the ML InSe, indicating the construction of type-II heterostructure for the InSe-GeTe HBL, which would promote the effective separation of holes and electrons, and provide a strategy to employ it as an electron–hole separator.

### 3.3. Biaxial Strain Effect on InSe-GeTe HBL

It is generally suggested that the mechanical strain is a simple method for modulating the electronic structures and optical properties of 2D materials, especially by employing biaxial strain [27,28,29,30,31]. Here, the effect of planar biaxial strain on the band structures, projected density of states (PDOS), and optical absorption spectra under different biaxial compressive and tensile strains has been systematically explored. The biaxial strain of the InSe-GeTe HBL is calculated by changing the lattice constant and expressed by the following formula, with the strain (ε) defined as

(5) $$\varepsilon =\frac{\left(a-a_{0}\right)}{a_{0}}\times 100\text{\%}$$

where a and $a_{0}$ are the strained and unstrained lattice constants, respectively.

**Figure 5:**
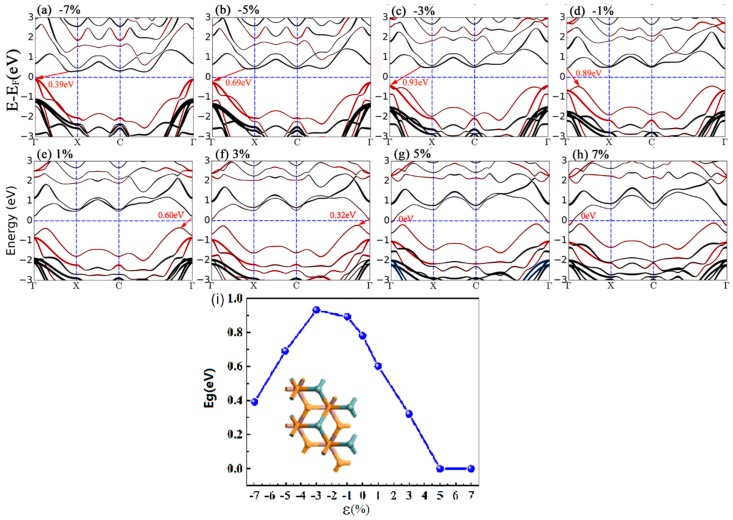
The band structures of the InSe-GeTe HBL under compressive strains of ε = (**a**) −7%, (**b**) −5%, (**c**) −3%, (**d**) −1%, and under tensile strains of ε = (**e**) +1%, (**f**) +3%, (**g**) +5%, (**h**) +7%. The bands drawn in red and black represent the bands dominated by GeTe and InSe monolayers, respectively. (Fermi level is set to 0 eV, and indicated by a dashed line). (**i**) Band gap values of the InSe-GeTe HBL as a function of biaxial strain.

**Figure 6:**
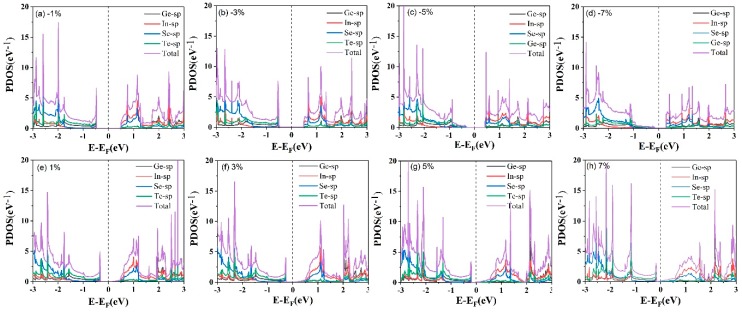
The calculated projected density of states (PDOS) of the InSe-GeTe HBL with biaxial strains of (**a**) −1%, (**b**) −3%, (**c**) −5%, (**d**) −7%, (**e**) 1%, (**f**) 3%, (**g**) 5%, and (**h**) 7%. The vertical black dashed line is the Fermi level.

Figure 5 depicts the fat band structures of the InSe-GeTe HBL under different compressive and tensile biaxial strains. First of all, it is clearly seen that the band gap of the InSe/GeTe HBL enlarges monotonically as the compressive strain varies from −7% to −3%, the largest band gap of 0.93 eV appears under the strain of −3%. Then the band gap declines slowly from 0.93 eV to 0.78 eV with the strain changed from −3% to 0%. Under the conditions of tensile strains, the band gap decreases monotonically from 0.78 eV to 0 eV, when the strain changes from 0% to 7%. It is worthy to note that the CBM moves down and goes through the Fermi level until the tensile strain exceeds 5%, causing the transformation of InSe-GeTe HBL from semiconducting to metallic characteristic. Moreover, Figure 5i shows the change trend of the band gap values as a function of strain more intuitively. The results indicate that the band structures of the InSe-GeTe HBL is expected to be tuned by applying biaxial strain. In addition, Figure 6 shows the calculated PDOS of the InSe-GeTe HBL under the compressive and tensile strains ranging from −7% to 7%. It is found that the CBM is primarily dominated by the In-sp and Se-sp, while the VBM is contributed by the Ge-sp, Te-sp, both under compressive strain and tensile strains for the InSe-GeTe HBL, which infers that the characteristic of type-II HBL maintains under strain conditions.

**Figure 7:**
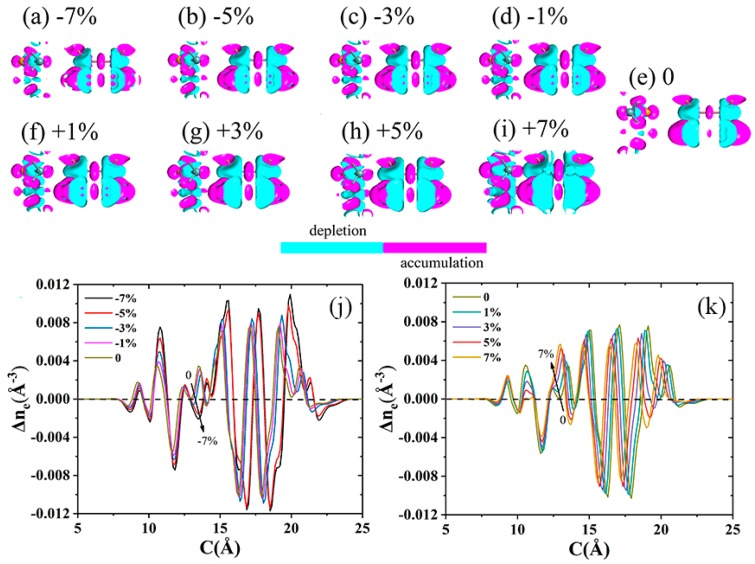
(**a**–**h**) 3D plot and (**j**,**k**) plane-averaged electron density difference Δne(c) along the c direction perpendicular to the interface for the InSe-GeTe HBL under the compressive strains of ε= −7%, −5%, −3%, −1%, and the tensile strains of ε = +1%, +3%, +5%, +7%. The plot of electron density difference for the InSe-GeTe without strain (ε = 0) is considered as a reference. The purple and blue regions denote the electron accumulation and depletion, respectively.

Figure 7 exhibits the electron density difference in two different manifestations (line graphs and atomic illustrations) for the InSe-GeTe HBL under different strains. The purple and blue regions denote the accumulation and depletion of electrons, respectively. It can be seen from the plane-averaged electron density difference that a slight charge redistribution occurs in the interface region. When the HBL is under the compressive strain of −7%, −5%, −3%, and −1%, the charge transfer amount from the GeTe to InSe monolayer by Mulliken (net charges) population analysis is 0.025|e|, 0.028|e|, 0.002|e|, and 0.005|e|, respectively. Unlike the condition of compressive strain, the amount of charge transfer from the GeTe to InSe monolayer under tensile strain is significantly enhanced, which are obtained as 0.032|e|, 0.058|e|, 0.072|e|, and 0.074|e|, corresponding to ε = 1%, 3%, 5%, and 7%, respectively. It is noted in Figure 7j,k that the amount of electron-transfer increases with the enhanced tensile strain, and reduces with the increased compressive strain. Moreover, the compressive strain can induce more pronounced electron transfer than that of the tensile strains in InSe-GeTe HBL. These results indicate that the strain would effectively tune the carrier concentration and charge distribution.

**Figure 8:**
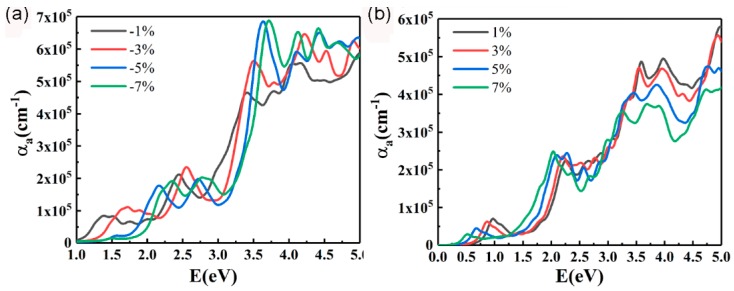
Optical absorption spectra of the InSe-GeTe HBL under (**a**) compressive and (**b**) tensile strains.

In order to study the effect of strain on the optical properties of InSe-GeTe HBL, the absorption spectra of the HBL under biaxial compressive and tensile strains are presented in Figure 8a and Figure 8b, respectively. It is proved that the absorption coefficients (*α_a_*) gradually increases with the enhancement of both compressive and tensile strains in the visible light range. Additionally, the absorption coefficients in the UV region from 250 (~5.0 eV) to 400 nm (~3.0 eV) are much larger that in the visible light region, indicating the strong UV absorption of the designed HBL. More specifically, a blue-shift of the absorption peak position for the HBL under compressive strain, and red-shift of the absorption peak position for the HBL under tensile strain with the increase of strain are obviously observed, which is consistent with the change of the band gap under different strains mentioned above. As a result, it is also demonstrated that the optical properties of the InSe-GeTe HBL could be modulated by employing biaxial strain.

## 4. Conclusions

In conclusion, we systematically investigate the electronic and optical properties of the InSe-GeTe vdW HBL under biaxial strains based on first-principles calculations. The most stable stacking mode of the InSe-GeTe HBL is optimized, which is demonstrated to be a construction of type-II hetero-junction, and is beneficial for separating electron-hole pairs. Moreover, the employment of biaxial strain exhibits prominent effect on modulating the electronic and optical properties of the designed InSe-GeTe HBL. Particularly, band structures and band gaps of the HBL could be tuned regularly under compressive and tensile biaxial strains. Improved optical absorption intensity in the visible wavelength region, and shift of absorption peak positions under different strains indicate that the designed InSe-GeTe HBL would be a promising candidate for optoelectronic applications.

## Figures and Tables

**Table 1:** First-principles calculation parameters of the Indium selenide-Germanium telluride (InSe-GeTe) heterobilayer (HBL).

Parameter	InSe-GeTe HBL
Monkhorst−Pack k point mesh	8 × 8 × 1
Mesh cutoff density (Hartree)	100
Forces tolerance per atom (eV/Å)	≤0.01
Stress error tolerance (eV)	≤10^−5^
Length of vacuum zong (Å)	≥20

**Table 2:** Calculated binding energies (E_b_), interlayer spacings (d), and band gaps (E_g_) of six different InSe-GeTe stacking structures.

Stacking Type	HBL Ⅰ	HBL Ⅱ	HBL Ⅲ	HBL Ⅳ	HBL Ⅴ	HBL Ⅵ
d (Å)	3.19	3.81	3.09	2.80	2.98	3.54
E_b_ (meV)	−67	−61	−66	−71	–70	−62
E_g_ (eV)	1.28	1.27	1.26	0.78	0.87	0.93
